# Could miRNA Signatures be Useful for Predicting Uterine Sarcoma and Carcinosarcoma Prognosis and Treatment?

**DOI:** 10.3390/cancers10090315

**Published:** 2018-09-06

**Authors:** Laura Gonzalez dos Anjos, Bruna Cristine de Almeida, Thais Gomes de Almeida, André Mourão Lavorato Rocha, Giovana De Nardo Maffazioli, Fernando Augusto Soares, Isabela Werneck da Cunha, Edmund Chada Baracat, Katia Candido Carvalho

**Affiliations:** 1 Laboratório de Ginecologia Estrutural e Molecular (LIM 58), Disciplina de Ginecologia, Departamento de Obstetricia e Ginecologia, Hospital das Clinicas da Faculdade de Medicina da Universidade de Sao Paulo, HCFMUSP, SP, BR Av. Dr Arnaldo 455, sala 4121, Cerqueira Cesar, São Paulo 05403-010, Brazil; lauragonzalezanjos@gmail.com (L.G.d.A.); bruc_10@hotmail.com (B.C.d.A.); tgalmeida.sp@gmail.com (T.G.d.A.); giovanamaffazioli@hotmail.com (G.D.N.M.); edmund.baracat@hc.fm.usp.br (E.C.B.); 2 Instituto Brasileiro de Controle do Cancer, SP, BR Av. Alcântara Machado, 2576 Mooca, São Paulo 05403-010, Brazil; 3 Hospital A C Camargo Cancer Center, SP, BR R. Tamandaré, 753 Liberdade, São Paulo 05403-010, Brazil; lavorato.andre@gmail.com (A.M.L.R.); fasoares@me.com (F.A.S.); iwerneck0210@gmail.com (I.W.d.C.); 4 Department of Pathology, Rede D’OR-São Luiz, Rua das Perobas, 344-Jabaquara, São Paulo 04321-120, Brazil; 5 National Institute for Science and Technology in Oncogenomics and Therapeutic Innovation, SP, BR R. Tamandaré, 753 Liberdade, São Paulo 05403-010, Brazil

**Keywords:** uterine sarcomas, carcinosarcoma, miRNA, oncomirs, miRNA expression

## Abstract

Changes in microRNA (miRNA) expression may lead to cancer development and/or contribute to its progression; however, their role in uterine sarcomas is poorly understood. Uterine sarcomas (US) belong to a rare class of heterogeneous tumors, representing about 1% of all gynecologic neoplasms. This study aimed to assess the expression profile of 84 cancer-related miRNAs and to evaluate their correlation with clinical pathological features. Eighty-two formalin-fixed paraffin-embedded (FFPE) samples were selected. In leiomyosarcoma (LMS), there was an association of lower cancer-specific survival (CSS) with the downregulation of miR-125a-5p and miR-10a-5p, and the upregulation of miR-196a-5p and miR-34c-5p. In carcinosarcoma (CS), lower CSS was associated with the upregulation of miR-184, and the downregulation of let-7b-5p and miR-124. In endometrial stromal sarcomas (ESS), the upregulation of miR-373-3p, miR-372-3p, and let-7b-5p, and the down-expression of let-7f-5p, miR-23-3p, and let-7b-5p were associated with lower CSS. Only miR-138-5p upregulation was associated with higher survival rates. miR-335-5p, miR-301a-3p, and miR-210-3p were more highly expressed in patients with tumor metastasis and relapse. miR-138-5p, miR-146b-5p, and miR-218-5p expression were associated with higher disease-free survival (DFS) in treated patients. These miRNAs represent potential prediction markers for prognosis and treatment response in these tumors.

## 1. Introduction

Uterine sarcomas (US) belong to a rare class of heterogeneous tumors comprising 3–7% of all malignancies in the uterus, representing about 1% of all gynecologic tumors [1]. In 2018, the American Cancer Society estimated 63,230 new cases of uterine body cancer in the United States, with 11,350 deaths estimated as being caused by these tumors [2].

Previously, the US were histologically classified into uterine carcinosarcoma (CS; also called malignant mixed Müllerian tumors (MMMT), uterine leiomyosarcoma (LMS), endometrial stromal sarcoma (ESS), and undifferentiated sarcomas [1,3]. Recently, CS have been reclassified as a metaplastic form of endometrial carcinoma; however, this tumor is still included in several studies of mesenchymal tumors and in the 2014 World Health Organization (WHO) US classification [4]. Currently, mesenchymal tumors are divided into LMS (63%), ESS (21%; usually divided into low-grade (LG-ESS) and high grade (HG-ESS), adenosarcomas (ADS, 5%), high grade undifferentiated sarcomas (5%), and other rare subtypes [5]. Here, we included CS samples for comparison with sarcomas samples considering their mesenchymal component.

US are characterized by occurrences of rapid progression, with high rates of local recurrence and distant metastasis, and poor prognosis. The overall survival rate is less than 50% in two years, even when diagnosed early [4]. The high lethality and poor prognosis are the main challenges for the optimal treatment of these tumors [6,7]. Despite their clinical relevance, few studies have been developed with regards to the molecular features involved in USs’ malignancy risk or biological behavior.

Epigenetic studies have demonstrated that microRNAs (miRNAs) are involved in multiple biological functions, including regulation of gene expression at the posttranscriptional level, cell proliferation, apoptosis, metabolism, angiogenesis, and others [8,9,10]. Thereby, miRNA differential expression caused by chromosomal alterations might lead to cancer development and/or its progression [10]. The molecular mechanism of miRNA and target gene interactions are not always clear, nor how altered miRNA patterns may directly cause cancer or be rather an indirect effect of changes in cellular phenotype. Nonetheless, miRNA expression patterns differ for specific tissues and differentiation states [11].

miRNA is a short non-coding RNA with around 19–27 nucleotides [12,13] that acts as a tumor suppressor (negatively regulating protein-coding oncogenes) or as an oncomir (repressing known tumor suppressors) and can affect cancer phenotypes [14]. Moreover, several reports have identified miRNA expression profiles, such as tumor signatures [14].

This molecule can control tumor metastasis through the divergent or convergent regulation of signaling pathways. The prognostic capacity of these non-coding RNAs in predicting human metastatic outcomes suggests an important role in cancer progression [15]. The molecular mechanisms of miRNAs and their roles in the physiological and pathological processes, as well as their correlation with prognosis and treatment prediction in patients with uterine mesenchymal tumors and CS, are still unknown. Attempting to identify predictor biomarkers based on the miRNA expression profile for US diagnosis, prognosis, or treatment led to the present study being conceived. This study aims to evaluate the expression profile of 84 oncomir sequences and to assess their expression profile correlation with the clinical pathological features of patients with LMS, CS, ESS, and ADS.

## 2. Results

For formalin-fixed paraffin-embedded (FFPE) sample analysis, we distributed the uterine sarcomas in four groups: The LMS group was composed of 37 patients with ages ranging from 32 to 91 years old (mean age: 56 ± 14.80 years old; data represents the mean ± SD); the CS group was composed of 23 patents with ages ranging from 54 to 87 years old (mean age: 67.30 ± 9.397 years old); the ESS group had 18 patients with ages ranging from 27 to 82 years old (mean age: 55.61 ± 15.41 years old); and two patients were included in the ADS group with ages ranging from 68 to 72 years old (mean age: 70 ± 2.828 years old). The LMS and CS groups differ in the age mean (*p* = 0.0048).

Clinical and anatomopathological features of evaluated patients in the study are described in Table 1 and Table 2.

**Figure 1:**
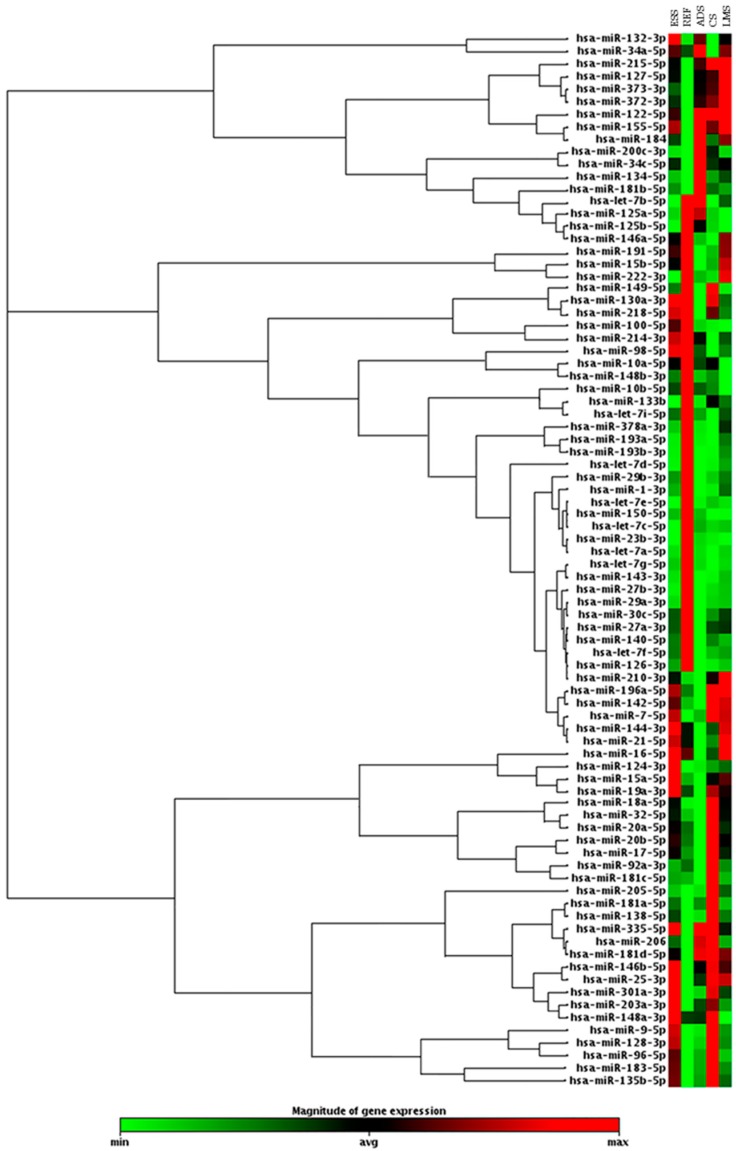
Clustergram analysis showing the expression profile of 84 oncomirs accessed in uterine leiomyosarcoma (LMS); uterine carcinosarcoma CS, endometrial stromal sarcoma (ESS), and adenosarcoma (ADS)*, using benign uterine tissue (Ref) as a reference for a normal gene expression profile (fold change (FC) expression cut-off values of +2 and −2). *ADS was not part of the statistical or subsequent analyses.

We performed a hierarchical clustering analysis of 84 miRNAs with an expression profile for each histological tumor group. Data analysis showed different expressions of 18 upregulated miRNAs in LMS samples, 22 in CS samples, 18 in ESS samples, and 15 in ADS samples. We found 6 downregulated miRNAs in cases of LMS, 9 in CS, 9 in ESS and 15 in ADS, using benign uterine samples as a reference for a normal gene expression profile (Figure 1).

Statistical analysis between the miRNA differential expressions and clinical pathological data of patients with LMS, CS and ESS showed that 59 miRNAs are significantly associated to clinical features. However, this analysis could not be performed given the limited number of samples in the ADS group. Among the significant associations found, we can highlight those related to the diagnosis, prognosis, and treatment. Shifts in the molecules’ expression of miR-301a-3p, miR-29a-3p, miR-29b-3p, and miR-193b-3p and in the tumor suppressor family let-7, miR-7-5p, miR-96-5p, miR-23b-3p, and miR-335-5p were associated to recurrence, metastasis, stages, and high histological degree (Table 3).

**Figure 2:**
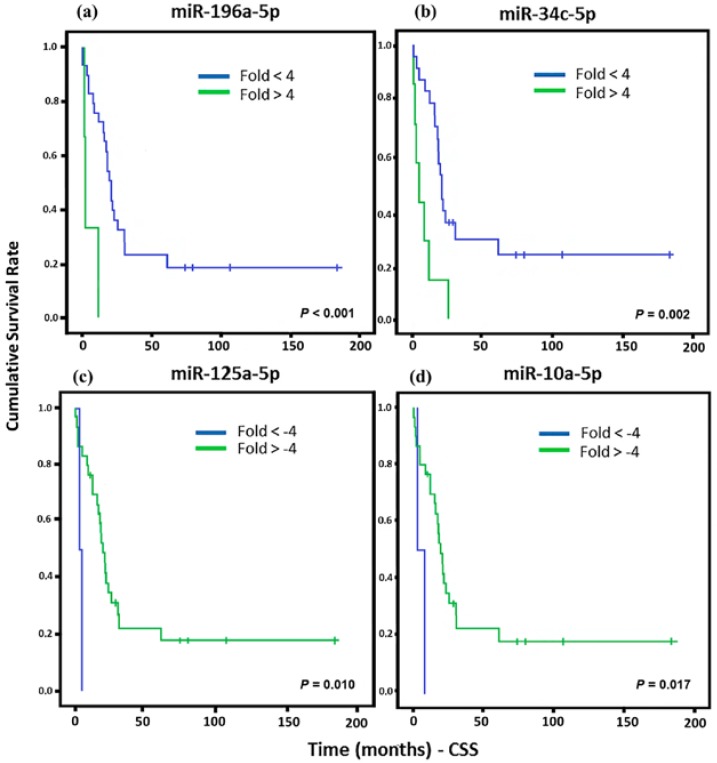
Kaplan Meier curves and estimates of cancer-specific survival (CSS) associated with miRNA expression in LMS patients. (**a**) miR-196a-5p showed a strong association with CSS (*n* = 32) (*p* < 0.001). (**b**) Association of miR-34c-5p expression with CSS (*n* = 31) (*p* = 0.002). (**c**) miR-125a-5p expression associated with CSS (*n* = 32) (*p* = 0.010). (**d**) miR-10a-5p expression was associated with CSS (*n* = 32) (*p* = 0.017).

In order to analyze the cancer-specific survival (CSS) of patients, we evaluate each miRNA that presented association with any tumor type. For all statistical analyses, we consider significantly different expressed miRNAs that were higher than the cut-off of >+4 and <−4. This cut-off was established to increase the accuracy of the results and, increase the chances of a biological validation. Significant associations of the downregulation of miR-125a-5p and miR-10a-5 with a lower CSS were observed in LMS, whereas miR-196a-5p and miR-34c-5p upregulation were associated to low CSS (Figure 2).

**Figure 3:**
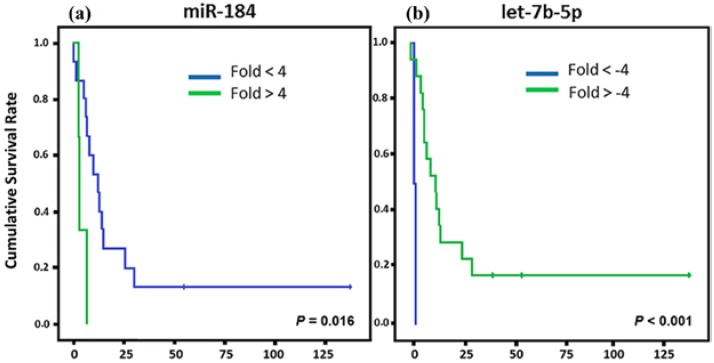

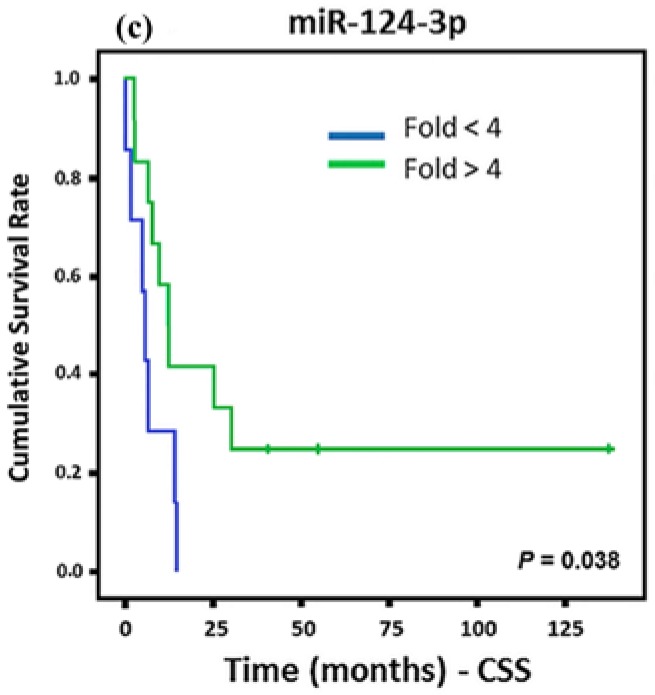
Kaplan Meier curves and estimates of CSS associated with miRNA expression in CS patients. (**a**–**c**) The expression profiles of miR-184 and miR-124-3p showed associations with CSS (*n* = 19) (*p* = 0.016 and *p* = 0.038, respectively) in both cases; while let-7b-5p exhibited a strong association with CSS (*n* = 19) (*p* < 0.001).

For the CS patients, we found that three miRNAs (miR-184, let-7b-5p, and miR-124-3p) showed association with CSS (Figure 3). miR-184 upregulation and let-7b-5p and miR-124-3p down regulation showed association with a lower CSS.

**Figure 4:**
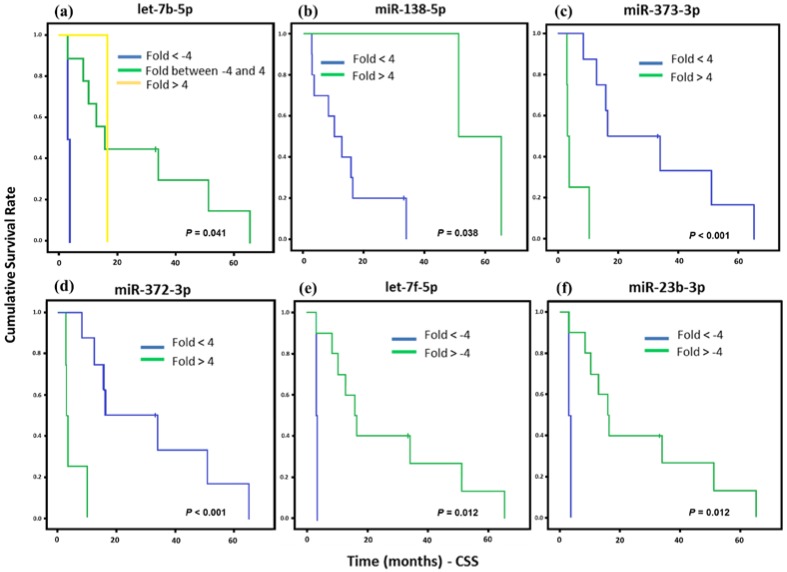
Kaplan Meier curves and estimates of CSS associated with miRNAs expression in high grade ESS (HG-ESS) patients (*n* = 12). (**a**) let-7b-5p expression associated with CSS (*p* = 0.041) both up and downregulation of this gene were associated to poorer prognosis. (**b**) Association of miR-138-5p expression with CSS (*p* = 0.038). (**c**,**d**) miR-373-3p and miR-372-3p were highly associated with CSS (*p* < 0.001). (**e**,**f**) let-7f-5p and miR-23b-3p expression were associated with CSS (*p* = 0.012).

The overall survival of ESS patients showed association with six miRNAs’ (let-7b-5p, miR-138-5p, miR-373-3p, miR-372-3p, let-7f-5p, and miR-23b-5p) differential gene expression. After that, the survival rates were assessed only for HG-ESS patients (12 cases) due to its poor prognosis and lower overall survival rates. A great number of ESS cases were present in our samples (18 samples), but six were classified as low histological grade and did not allow for correlation analysis between miRNA profile and survival rates (Figure 4). In these samples, miR-373-3p and miR-372-3p up-regulation showed correlation with a lower CSS, as well as let-7f-5p and miR-23b-3p downregulation. Only miR-138-5p upregulation was associated with higher survival.

**Figure 5:**
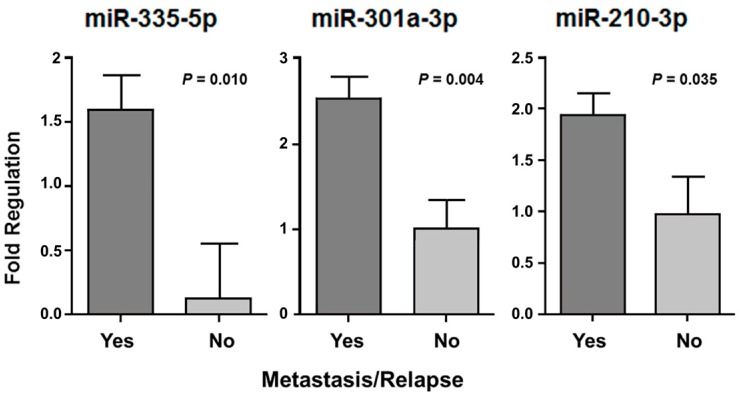
The miR-335-5p, miR-301a-3p, and miR-210-3p with significant expression difference in patients who had undergone adjuvant treatment and presented metastasis or tumor relapse, independent of the tumor histological type.

We assessed the disease-free survival (DFS) in 60 patients who had undergone adjuvant treatment (chemotherapy and/or radiotherapy), considering metastasis and/or relapse occurrence. Three miRNAs (miR-335-5p, miR-301a-3p and miR-210-3p) presented differences of expression when we compared two groups of patients: those who had adjuvant treatment and developed metastasis or tumor recurrence (*n* = 41) with patients who also had adjuvant treatment but did not develop metastasis or relapse (*n* = 13) (Figure 5). The molecules that appeared significantly associated in this analysis presented a higher expression in patients with metastatic tumors or who presented recurrence (*p* < 0.05).

**Figure 6:**
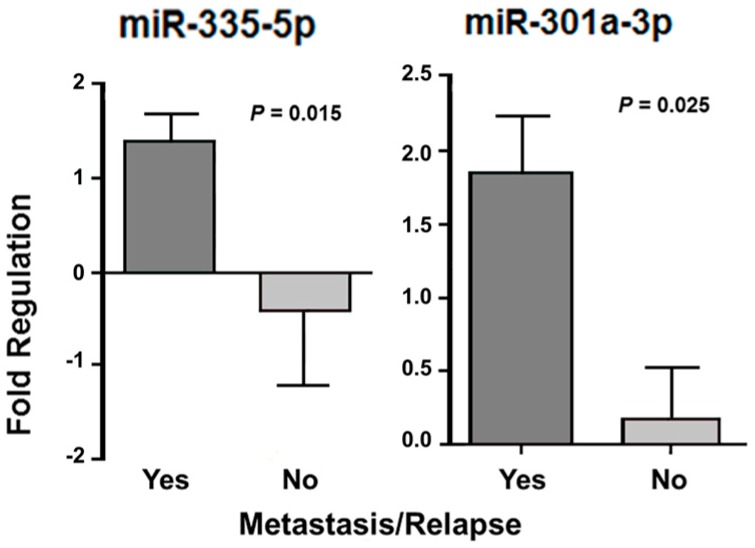
miRNAs with significant differential expression according to the presence of metastasis and/or relapse after treatment in patients with LMS.

Twenty-one LMS patients had metastasis and/or relapse and five did not show tumor progression. In the CS patients, nine presented metastasis and/or relapse, but in five patients, the tumor had not progressed. Almost all ESS patients (*n* = 13) had metastasis and/or relapse, whereas two patients did not have the tumor spread. In these 13 cases, four were classified as low-grade, where two of them had metastasis or relapse. All others were classified as high grade with presence of metastasis or relapse. One of two patients with ADS had tumor metastasis and/or relapse. In the association analysis of the miRNA expression and metastasis and/or relapse in LMS and CS patients, there was a significant differential expression of miR-335-5p (*p* = 0.015) and miR-301a-3p (*p* = 0.025) only in LMS. CS analysis was performed, but with no statistical significance. The reduced sample number (*n* = 2) was a limitation for the statistical analysis in ESS patients without metastasis and/or relapse (Figure 6).

**Figure 7:**
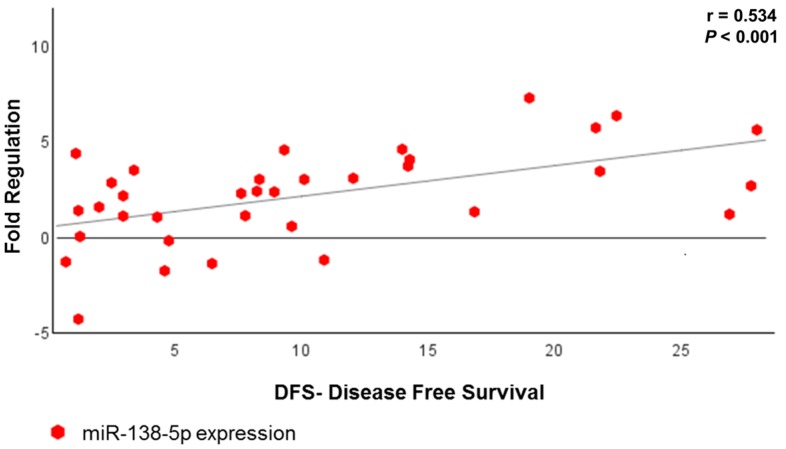
Scatter plot showing the association between miR-138-5p expression and DFS in all US patients who underwent adjuvant treatment.

**Figure 8:**
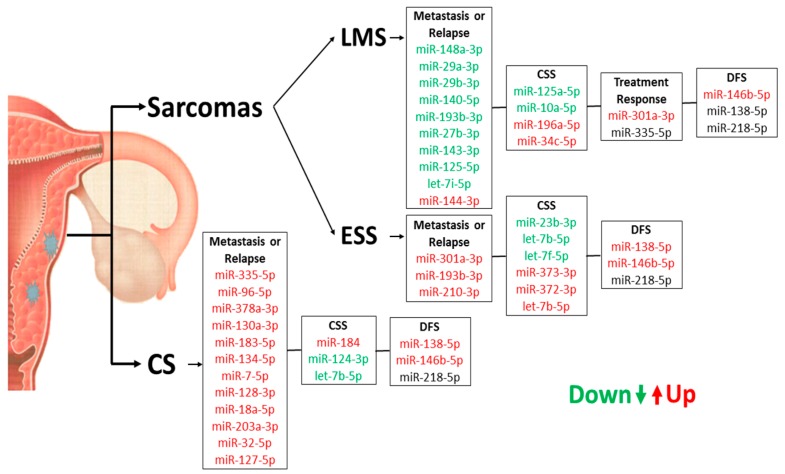
Summary of miRNA expression profiles in US and CS samples concerning their prognostic role for patients.

We performed a correlation analysis with all tumor groups that showed a positive association between three miRNAs (miR-138-5p, miR-146-5p and miR-218-5p) with DFS (Table 4); of these, the miR-138-5p displayed a strong correlation with DFS (*p* < 0.001) (Figure 7). Figure 8 shows briefly the expression of the 48 miRNAs correlated to metastasis (or relapse), CSS, treatment response and DFS.

**Figure 9:**
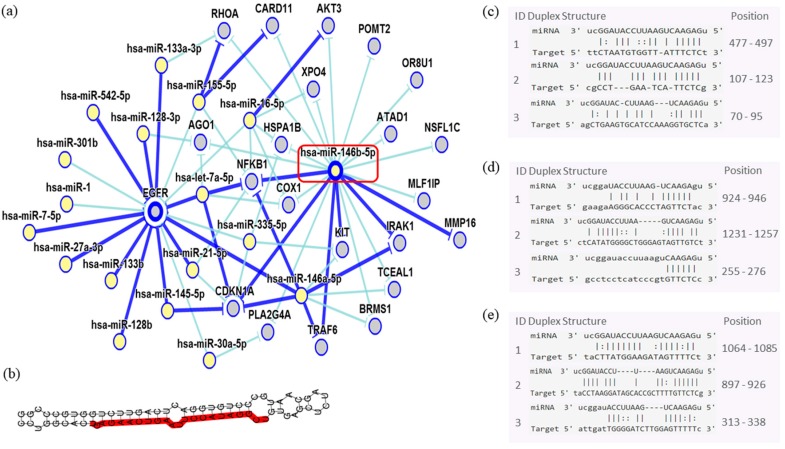
Interaction network of miR-146b- 5p. (**a**) The mRNA showed a strong modulation of *NFKB1* and *CDKN1A*, and an indirect interaction with *EGFR*. (**b**) Molecular structure: pre-miRNA hairpin of miR-140b- 5p, second structure of pre-miRNA, and, in red, mature sequence 9|UGAGAACUGAAUUCCAUAGGCU|30. (**c**–**e**) miRNA sequence of target interactions with *NFKB1, CDKN1A* and *EGFR*, respectively, showing the position in the gene sequence. Dark blue shows strong evidence (reporter assay, Western blot, qRT-PCR, or qPCR), and light blue shows other evidence.

**Figure 10:**
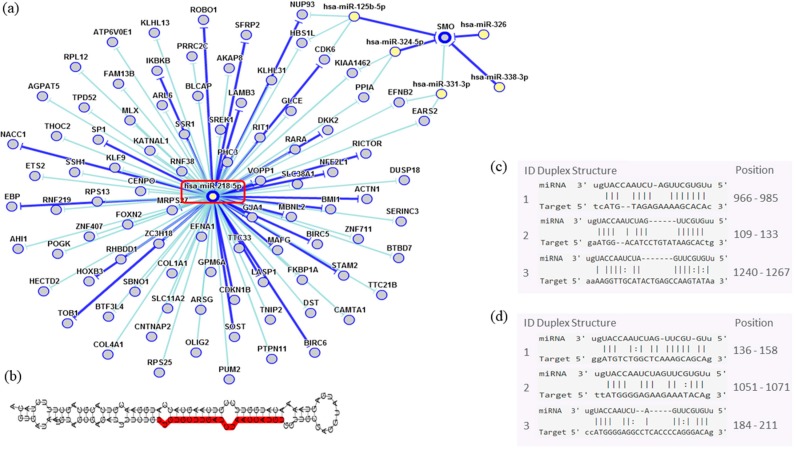
Interaction network of miR-218-5p. (**a**) The mRNA showed a strong modulation of *CDKN1A* and an indirect interaction with *SMO*. (**b**) Molecular structure: pre-miRNA hairpin of miR-218-5p, second structure of pre-miRNA, and, in red, mature sequence 25|UUGUGCUUGAUCUAACCAUGU|45. (**c**,**d**) miRNA sequence of target interactions with *CDKN1A* and *SMO*, respectively, showing the position in the gene sequence. Dark blue shows strong evidence (reporter assay, Western blot, qRT-PCR or qPCR), and light blue shows other evidence.

**Figure 11:**
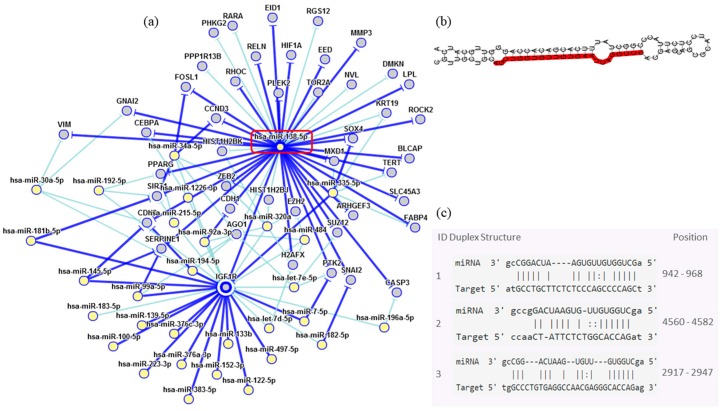
Interaction network of miR-138-5p. (**a**) The mRNA showed a strong modulation of *IGF1R*. (**b**) Molecular structure: pre-miRNA hairpin of miR-218-5p, second structure of pre-miRNA, and, in red, mature sequence 23|AGCUGGUGUUGUGAAUCAGGCCG|45. (**c**) miRNA sequence of target interactions with *IGF1R* showing the position in the gene sequence. Dark blue shows strong evidence (reporter assay, Western blot, qRT-PCR or qPCR), and light blue shows other evidence.

In order to assess the miRNAs’ network of genetic interactions and their potential targets, the miR-146b-5p, miR-218-5p and miR-138-5p were selected based on their association with DFS in US and carcinomas patients who had undergone adjuvant treatment (Figure 9, Figure 10 and Figure 11).

Through the bibliographic research of miRNAs validation studies with their respective target genes and with the aid of the miRTarBase platform (http://mirtarbase.mbc.nctu.edu.tw/php/index.php), the main interaction network among these molecules was established. Relevant interactions were found between the miR-146b-5p and the *NFKB1* and *CDKN1A* genes. This miRNA was also indirectly correlated to the *EGFR* gene. miR-218-5p was directly associated to the *CDKN1A* gene, and indirectly to the *SMO* gene. Lastly, miR-138-5p was related to the *IGF1R* gene. Genes directly regulated for these miRNAs were related to transcriptional regulation, growth factors, cell cycle regulation and encoded protein transduces signals. These results are encouraging for future research aiming to discover the biological effects of these interactions.

## 3. Discussion

This study corroborates the high-aggressive profile of US with a greater propensity to metastasize, as previously demonstrated in several studies [16,17,18,19]. These tumors are, therefore, associated with high rates of mortality. In the current study, 59% of evaluated patients were more than 50 years old at diagnosis. As seen in the literature [6,20,21], the majority of patients had abnormal vaginal bleeding, especially at the post-menopausal stage. Hysterectomy is considered the standard surgical treatment for US and CS [22,23], and was performed in 98% of patients. Sixty-five percent of patients were diagnosed with metastasis, with more than half (67.3%) spread out to distant organs. In addition, most patients died (70%). Naaman et al. [24] described that the US overall survival rate is low: 50–70% for patients with stage I, and 0–20% only for other stages.

Elucidating the role of miRNAs in tumor behavior and development was also an aim of the present study. In spite of the scarcity and the heterogeneity of these tumors, we attempted to find specific tumoral signatures. To the best of our knowledge, there are no adequate biomarkers available yet for the diagnosis or correct evaluation of these neoplasms. miRNAs have been studied in uterine tumors, and aberrant expression patterns were identified in several sarcoma types [25]. Clinical features such as age, stage, tumor size, presence or absence of necrosis, and mitotic index [6] along with miRNA differential expression profiles might be important to determining these tumors’ prognoses. Here, we found miRNA expression profiles were associated with the CSS of patients. miR-196a-5p, miR-34c-5p, miR-125a-5p, and miR-10a-5p expressions were associated with CSS in LMS women. Additionally, the miR-184, miR-124-3p and let-7b-5p showed an association between CSS and CS. Differential expressions of let-7b-5p, miR-138-5p, miR-373-3p, miR-372-3p, let-7f-5p, and miR-23b-3p were associated with CSS in HG-ESS. Due to the limited number of ADS samples, survival analysis for this group was not performed. Although the mentioned miRNAs may represent potential prognostic biomarkers, future biological validations are necessary for their precise role establishment in the uterine tumors.

Furthermore, associations between miRNA expression patterns with clinical features led us to a better understanding of how these markers are related to the greater or lesser survival rate of patients. In addition, our findings showed strong associations between miRNA expression (down or upregulated) and patients’ clinical features, such as recurrence, relapse, metastasis and survival rates. Basic miRNA expression profiling is proving to be clinically relevant to cancer diagnosis, progressions and outcome [11,26,27]. The association of miRNA expression levels with anatomopathological and clinical data may be relevant as a diagnostic biomarker for identifying or differentiate US.

Kowalewska et al. 2013 [28] evaluated the expression profile of miRNAs in LMS, ESS, CS, and ADS. Our study corroborates many of the differences in miRNA expression profiles found in this study. Both studies found differences in the expression of the miR-23, miR-1, let-7f, and let-7c in ESS in relation to benign samples. Another similar result was associated with mixed tumors. Both studies showed the downregulation of miR-1, let-7c, miR-133b, let-7b, miR-143, let-7a, let-7d, let-7e, let-7g, miR-222, let-7i, and miR-214 in relation to the benign tissue. The upregulation of the miR-206 was also observed in the two studies. The miR-206 has *NOTCH3* as target gene. The pathogenic effects caused when the *NOTCH* signaling pathway is modified were already described in other sarcomas [28,29].

A recent molecular study analyzed 381 miRNAs in LMS and ESS, showing different expression in 94 miRNAs. After validation experiments, seven miRNAs (miR-15b, miR-21, miR-23b, miR-25, miR-145, miR-148b e miR-195) were upregulated in ESS when compared to primary LMS. Among these molecules, only the expression of miR-25 presented a similar profile in our analysis [30]. Changes in miR-25 expression have already been associated with lymph node metastasis in osteosarcoma [31].

Shi et al. 2009 [32] found a significant correlation between endogenous *HMGA2* levels and let-7 expression in uterine LMS. In this study, it has been verified that treatment with exogenous let-7 significantly reduced cell proliferation through the repression of *HMGA2*. We evaluated the expression of eight molecules of the let-7 family and all were shown to be altered, mainly let-7a-5p (fold: −5.41).

All studies have found limitations in sample size, due to tumor rarity, but the observed similarities can be significant for future research. These differences may have occurred due to sample size differences, tumor heterogeneity and differences in the specific population.

Global gene expression patterns were compared in primary and metastatic LMS. The existence of a unique genetic signature was confirmed for these conditions. A high expression of *TNNT1*, *FOLR3*, *TDO2*, *CRYM*, *GJA1*, *TSPAN10*, *THBS1*, *SGK1*, *SHMT1*, *EGR2* and *AGT* genes in metastasis were displayed [33]. Through interaction analysis of miRNA and their target genes in the data bank, we observed that many miRNAs analyzed in our study are associated with recurrence, relapse and metastasis, regulating mentioned genes from Davidson et al.’s [33] research. We found that miR-29b-3p and miR-29a-3p regulate *SGK1*; miR-335-5p, miR-125b-5p and miR-193b-3p regulate *SHMT1*; miR-130a-3p regulates *GJA1*; and miR-18a-5p and let-7i-5p regulate *THBS1*. This analysis indicates that the deregulation of these molecules can be associated with more aggressive tumor phenotypes.

Almost one-third of miRNAs located in a small region of chromosome 14q32 are significantly altered in CS tissues compared to benign endometrium [34]. Analogously to the study of Devor et al. [34], miR-127-5p, which is associated with the presence of recurrences in CS patients in our study, displayed expression alterations in this tumor type. Data indicate that a specific deregulation contributes to a unique histology and worse prognosis in CS.

In the ESS, the miR-210-3p seems to be specifically related to metastasis in our analysis. Our data revealed that the expression profile of three miRNAs miR-146b-5p (*p* = 0.032) miR-218-5p (*p* = 0.048) and miR-138-5p (*p* < 0.001) correlated to DFS in patients that had undergone adjuvant treatment. The miR-146b-5p seems to strongly modulate critical genes such as *NFKBI* transcriptional factor that are involved in inflammatory responses and may be a possible mediator of doxorubicin effects in sarcomas [35]. miR-146b exerts potential effects on *EGFR* by the *NFKBI* signaling pathway. *EGFR* was described as immunolocalized of its proteins in the cytoplasm of smooth-muscle cells in the leiomyoma and matched myometrium [8,36]. Strong interactions might be seen between miR-146b and *CDKN1A*, which is an important regulator of cell cycle progression at G1 and can act as a positive regulator of senescence-like terminal proliferation arrest, but its function seems neither sufficient nor absolutely required for a treatment response to doxorubicin in tumor cells, especially soft tissue sarcoma [35].

Potential tumor suppressor miR-218-5p strongly regulates the *CDKN1B*, which is responsible for cell cycle progression control. This gene is widely studied in other sarcoma types such as Kaposi’s sarcoma and osteosarcoma, which exhibit aberrant *CDKN1B* subcellular localization [37,38]. In addition, an indirect miR-218 interaction with *SMO* may be seen; however, the effects of this signaling pathway of are not well understood. Garcia et al. [39] evaluated the smoothened, frizzled class receptor (SMO) protein in LMS and observed that there was in unusual increased protein expression.

Although its function is uncertain, the miR-138-5p is an important *IGF1R* regulator. *IGF1R* was detected in the uterus and its expression levels were significantly higher in uterine fibroids. There is a hypothesis that the fibroids’ transformation is the main cause of LMS [3,8]. The main function of the *IGF1R* as a survival factor is to inhibit apoptosis, being able to increase the tumorigenic potential under some conditions, which protects them from programmed cell death when over-expressed [40]. There is a clue that when correlated with DFS, the expression of miR-146b-5p, miR-218-5p and miR-138-5p may have a role in adjuvant therapy response in US patients. Further studies are necessary to elucidate its effects in US.

New therapeutic strategies focus on recovering tumor suppressor proteins and inhibiting oncogenic ones by restoring the miRNAs that are down-regulated and silencing the miRNAs that are up-regulated [8,41]. The inhibition or delivery of miRNA may provide a highly potent means to modulate the cancer process while avoiding unwanted toxic effects in adjacent non-cancerous tissue [42]. Considering the fact that miRNAs are endogenous regulators, mechanisms that would attenuate their deregulation are likely to occur in cells, decreasing the risk of the off-target effects after their therapeutic delivery. The ability of a single miRNA to act in multiple pathways seems to decrease the possibility of developing resistance to the treatment [27].

## 4. Materials and Methods

### 4.1. Samples

This study was conducted in accordance with the Declaration of Helsinki and it was approved by the Research Ethics Committee of the Faculdade de Medicina da Universidade de Sao Paulo (FMUSP) under protocol number No. 143/11, from 2014 to 2016. Subjects gave their informed consent for inclusion before they participated in the study.

In order to compare the miRNA expression profile in different histological types of US, we selected 82 FFPE human samples (including 37 samples of LMS, 23 of CS, 18 of ESS, 2 of ADS and two benign uterine tissue myometrium). Patient samples with benign uterine tissue were only used as references (Ref) of normal gene expression; no statistical analyses were performed including them. All samples were stored at the Molecular and Structural Gynecology Laboratory of University of Sao Paulo Medical School (FMUSP). Samples were provided by the Instituto Brasileiro de Controle do Cancer, Hospital Santa Marcelina, AC Camargo Cancer Center and Disciplina de Ginecologia do Hospital das Clinicas da Faculdade de Medicina de São Paulo.

Clinical data were collected from patient charts and analyzed. The staging was performed according to the latest edition of the International Federation of Gynecology and Obstetrics (FIGO)-2009 [43]. Histological grade classification was based on nuclear polymorphism and mitotic index (Diagnostic Pathology Gynecological, 2014) [1].

### 4.2. MicroRNAs Expression Profile Analysis

The Qiagen miRNeasy FFPE Kit (Qiagen, Hilden, Germany) was used to extract miRNA from FFPE tissues. All samples were quantified by the spectrophotometer NanoDrop 2000 (Thermo Scientific^TM^, Fremont, CA, USA) and cDNA synthesis (reverse transcription) was carried out using the miScript II RT Kit (Qiagen, Hilden, Germany) following the manufacturer’s instructions.

Subsequently, the quantitative real-time PCR (qRT-PCR) was carried out using the miScript SYBR^®^ Green PCR Kit (Qiagen, Hilden, Germany) with the Human Cancer Pathway finder miRNA PCR Array MIHS-102Z-Qiagen 96 wells plate (Qiagen, Hilden, Germany), which contains 84 miRNA sequences. Levels of miRNA expression were determined by the 6 housekeeping snRNAs (SNORD-61, SNORD-68, SNORD-72, SNORD-95, SNORD-96A, and RNU6-2) for normalization, 2 extraction controls, 2 reverse transcription controls, and 2 PCR positive controls. Reactions were incubated for 15 min at 95 °C followed by 40 cycles for 15 s at 94 °C, 30 s at 55 °C, and 30 s at 70 °C in the ABI 7500 Real-Time PCR (Thermo Scientific^TM^, Fremont, CA, USA). All data of relative expression were analyzed with the comparative cycle threshold method by ΔΔCt [44]. The data are presented as fold regulation, which indicates expression results in a biologically meaningful form and easy to read. Values < 0 (zero) are negative (downregulated) and > 1 (one) are positive (upregulated) in relation to a reference sample. Here, we established the cut off value of ±4 to increased biological relevance and functional validation chances of these molecules. In silico analysis was performed to identify the genetic interactions network related to tumorigenesis [45] as described previously by our group [8].

### 4.3. Statistical Analysis

Statistical analysis was performed using GraphPad Prism version 6.07 (GraphPad Software, San Diego, CA, USA), and SPSS version 13.0 (Chicago, IL, USA) for Windows. Continuous data were analyzed for normality. Students’ *t*-test or ANOVA were used for between-group comparisons for parametric variables and the Mann Whitney U or Kruskal Wallis test were used for non-parametric variables.

Associations between miRNA expression and clinical pathological features were analyzed by Fisher’s exact test. Among the clinical pathological variables analyzed were age, main symptoms, oral contraceptive use, menopause, hormone replacement therapy, smoking, surgical treatment, adjuvant treatment, persistence or tumor recurrence, local or distant metastasis, degree of tumor differentiation, current status and staging. For survival curves, we used the Kaplan Meier analysis and its significance was established by log-rank-test. Survival rates were calculated based on the time or period, in months, between the date of surgery and the date of death or the date of last information. CSS was calculated in this study to exclude deaths from causes other than cancer. DFS was evaluated based on the time period, in months, between the date of surgery and the date of diagnosis of the first metastasis.

Relapse was calculated based on the time or period between the date of the anatomopathological examination and the diagnosis of recurrence of the disease over a period of more than 6 months. The persistence of the disease was considered in cases where they presented evidence of the disease already at the anatomopathological diagnosis or until, at most, 6 months from the diagnosis. Metastasis cases were considered to be those that presented distant or locoregional disease, independent of the period. Statistical significance was set at *p* < 0.05.

## 5. Conclusions

We found miR-146b-5p, miR-218-5p and miR-138-5p with higher expression in patients who underwent treatment. Concerning metastasis and recurrence, miR-210-3p was associated with ESS metastasis; miR-127-5p was related to CS relapse; and two members of the miR-29 family (29a-3p and 29b-3p) were associated with aggressive phenotypes in LMS. Besides this, the upregulation of miR-196a-5p, miR-34c-5p, miR-373-3p, miR-372-3p and downregulation of let-7b-5p were strongly associated with a lower CSS. Further studies are ongoing to determine the potential application of these molecules for the specific diagnosis, prognosis and treatment of these tumors.

## Figures and Tables

**Table 1:** Clinical pathological features of cancer patients (*n* = 80) *.

Variables	Categories	*n* (%)
Age	>50 years	59 (74)
	≤50 years	21 (26)
	N. A.	0 (0)
Main complaint	Bleeding	48 (60)
	Pelvic Pain	18 (23)
	N. A.	14 (17)
Menopause	Yes	54 (67)
	No	11 (14)
	N. A.	15 (19)
Contraceptive	Yes	3 (4)
	No	42 (52)
	N. A.	35 (44)
Hormone replacement therapy (HRT)	Yes	2 (3)
	No	49 (61)
	N. A.	29 (36)
Smoking	Yes	3 (4)
	No	30 (37)
	N. A.	47 (59)
Surgical treatment	Yes	78 (98)
	No	2 (2)
Follow up	Average	36 months
	Range	1 to 183 months
Metastasis	Yes	52 (65)
	No	21 (26)
	N. A.	7 (9)
Death	Yes	55 (69)
	Alive	15 (19)
	N. A.	10 (12)

* N. A: not available.

**Table 2:** Clinical and anatomopathological aspects according to the histological type of neoplasia (*n* = 80) *.

Variables	Categories	LMS *n* (%)	CS *n* (%)	ESS *n* (%)	ADS *n* (%)
Clinical Stage	I	10 (27)	4 (17.4)	7 (38.9)	2 (100)
	II	10 (27)	2 (8.7)	3 (16.7)	0 (0)
	III	5 (13.5)	11 (47.8)	7 (38.9)	0 (0)
	IV	12 (32.5)	6 (26.1)	1 (5.5)	0 (0)
	N. A.	0 (0)	0 (0)	0 (0)	0 (0)
Histological Grade	High	27 (73)	23 (100)	13 (72.2)	1 (50)
	Low	10 (27)	0 (0)	5 (27.8)	1 (50)
	N. A.	0 (0)	0 (0)	0 (0)	0 (0)
Recurrence	No	6 (17)	8 (35)	3 (17)	1 (50)
	Persistence	12 (32)	6 (26)	7 (39)	0 (0)
	Relapse	15 (40)	5 (22)	7 (39)	0 (0)
	N. A.	4 (11)	4 (17)	1 (5)	1 (50)
Metastasis	Locoregional	12 (5)	2 (9)	3 (17)	0 (0)
	Distant	15 (55)	9 (39)	11 (61)	0 (0)
	N. A.	10 (40)	12 (52)	4 (22)	2 (100)
Adjuvant Treatment	No	10 (27)	5 (21.7)	5 (27.8)	0 (0)
	RT	11 (29.7)	8 (34.8)	6 (33.3)	2 (100)
	CT	11 (29.7)	3 (13.1)	4 (22.2)	0 (0)
	CT+RT	5 (13.6)	7 (30.4)	3 (16.7)	0 (0)
	N. A.	0 (0)	0 (0)	0 (0)	0 (0)
Status	Alive	7 (18.9)	3 (13)	4 (22.2)	1 (50)
	Death	26 (70.3)	16 (70)	13 (72.2)	0 (0)
	Loss of follow-up	4 (10.8)	4 (17)	1 (5.6)	1 (50)
	N. A.	0 (0)	0 (0)	0 (0)	0 (0)

RT: radiotherapy; CT: chemotherapy; LMS: uterine leiomyosarcoma; CS: carcinosarcoma; ESS: endometrial stromal sarcomas; ADS: adenosarcoma.

**Table 3:** Association between the expression of the microRNAs (miRNAs) and the clinical pathological features (*n* = 80) ^c^.

Clinical Variable	LMS		CS		ESS	
	miRNA	*p*	miRNA	*p*	miRNA	*p*
Age (over 50 years old)	220c-3p ^a^ 29a-3p ^b^	0.049 0.033	-	-	-	-
Main complaint—Bleeding	-	-	-	-	142-5p ^a^ 148b-3p ^a^ 214-3p ^a^	0.031 0.016 0.016
Menopause	148a-3p ^a^ 7-5p ^a^ 32-5p ^a^	0.022 0.023 0.030	30c-5p ^b,^* 148a-3p ^b^ 100-5p ^b^ let-7i-5p ^b,^*	0.001 0.004 0.034 0.001	335-5p ^b^	0.025
Oral contraceptive	-	-	-	-	132-3p ^b^ 34c-5p ^b^	0.005 0.006
HRT	-	-	-	-	125a-5p ^b^ 222-3p ^b^ 205-5p ^b^	0.039 0.039 0.019
Smoking	-	-	125a-5p ^a^ 30c-5p ^a^ 135b-5p ^a^ let-7i-5p ^a^ 27b-3p ^a^ 29a-3p ^a^ let-7f-5p ^a^ 34a-5p ^a^ 25-3p ^a^ 125b-5p ^a^ 23b-3p ^a^ 335-5p ^a^ 21-5p ^a^	0.018 0.018 0.011 0.006 0.011 0.044 0.018 0.011 0.007 0.028 0.028 0.044 0.029	27b-3p ^a^ 143-3p ^a^	0.006 0.038
Patients who died	96-5p ^b^ 132-3p ^b^ 183-5p ^b,^* 124-3p ^b^	0.023 0.026 0.001 0.024	122-5p ^a^	0.025	29b-3p ^b^ 206 ^b^	0.027 0.026
Presence of relapse	148a-3p ^a^ 29b-3p ^a^ 301a-3p ^b^ 29a-3p ^a^ 144-3p ^b^	0.021 0.008 0.027 0.040 0.020	335-5p ^b^ 96-5p ^b^ 378a-3p ^b^ 130a-3p ^b^ 183-5p ^b^ 134-5p ^b^ 7-5p ^b^ 128-3p ^b^ 18a-5p ^b^ 203a-3p ^b^ 32-5p ^b^	0.050 0.008 0.028 0.046 0.010 0.016 0.040 0.026 0.033 0.020 0.008	301a-3p ^b^	0.041
			127-5p ^b^	0.045		
Presence of metastasis	140-5p ^a^ 193b-3p ^a^ let-7i-5p ^a^ 27b-3p ^a^ 143-3p ^a^ 125b-5p ^a^	0.002 0.002 0.034 0.029 0.004 0.025	-	-	193b-3p ^b^ 210-3p ^b^	0.015 0.044
Stage I	-	-	-	-	-	-
Stage II	-	-	335-5p ^a,^* 218-5p ^a^	0.001 0.014	23b-3p ^a^	0.017
Stage IV	-	-	-	-	7-5p ^b^	0.014
High histological grade	let-7a-5p ^a^ 125a-5p ^b^ 222-3p ^b^ 148b-3p ^b^ 92a-3p ^b^ let-7b-5p ^a^ 205-5p ^b^ 20a-5p ^b^ 30c-5p ^b^ let-7g-5p ^a^ let-7c-5p ^a^ let-7e-5p ^a^ let-7f-5p ^b^ 218-5p ^b,^* 100-5p ^b^ 155-5p ^b^ 1-3p ^b^ 191-5p ^b^ let-7d-5p ^b^ 15b-5p ^b^ 98-5p ^b^ 25-3p ^b^ 128-3p ^b^ 143-3p ^b^ 19a-3p ^b^ 23b-3p ^b^ 16-5p ^b^ 17-5p ^b^ 18a-5p ^b^ 27b-3p ^b^	0.008 0.047 0.040 0.050 0.029 0.009 0.010 0.008 0.031 0.005 0.029 0.014 0.045 0.001 0.044 0.012 0.011 0.024 0.010 0.002 0.002 0.010 0.041 0.047 0.031 0.017 0.033 0.034 0.049 0.020	184 ^a,^*	0.001	-	-

^a^ miRNAs downregulated associated with the clinical variable (*p* < 0.05); ^b^ miRNAs upregulated associated with the clinical variable (*p* < 0.05); ^c^ Some sample data were not available; * *p* < 0.001.

**Table 4:** Correlation analysis between miRNA and DFS.

miRNA	r *	*p*
miR-146b-5p	0.358	0.032
miR-218-5p	0.331	0.048
miR-138-5p	0.534	<0.001

* Pearson correlation coefficient (r-value measures the intensity and direction of the linear connection between two quantitative variables).
